# Characteristic waffle-like appearance of gastric linitis plastica: A case report

**DOI:** 10.3892/ol.2014.2688

**Published:** 2014-11-07

**Authors:** EMIKO MAEDA, MAKOTO ORYU, JOJI TANI, HISAAKI MIYOSHI, ASAHIRO MORISHITA, HIROHITO YONEYAMA, HIDEKI KOBARA, HIROHITO MORI, TSUTOMU MASAKI

**Affiliations:** Department of Gastroenterology and Neurology, Kagawa University Faculty of Medicine, Kagawa 761-0793, Japan

**Keywords:** linitis plastica, scirrhous gastric cancer, diffuse type cancers, signet ring cell adenocarcinoma, waffle-like appearance

## Abstract

Linitis plastica is a gastric cancer of diffuse histotype that presents in the fundic gland area, and is characterized by thickening of the stomach wall and deformation of the stomach, resulting in a leather bottle-like appearance. A 66-year-old female was admitted to Kagawa University Hospital (Kagawa, Japan) with epigastric pain. X-ray examination revealed reduced gastric distension and deformation of the stomach, which exhibited a leather bottle-like appearance. Endoscopy indicated a depressed lesion in the gastric antrum, and abnormal folds, which crossed to form a waffle-like appearance in the upper gastric body. Analysis of biopsy specimens from the depressed lesion revealed a poorly differentiated adenocarcinoma. Morphological changes in the gastric folds indicated that the tumor had invaded the upper gastric body, therefore, a total gastrectomy was performed. Subsequent pathological findings demonstrated that the tumor had spread from the primary lesion to the upper gastric body. Therefore, the present report recommends that the diagnosis of the spread of linitis plastica-type gastric cancer should include assessments of the primary lesion, as well as evaluation of morphological changes in the gastric folds.

## Introduction

Linitis plastica is defined as a gastric cancer of diffuse histotype (1) that presents in the fundic gland area, and is characterized by thickening of the stomach wall resulting in deformation of the stomach and a leather bottle-like appearance. Since the majority of linitis plastica patients are diagnosed at an advanced stage of the disease, clinical outcomes are commonly observed, regardless of the extent or type of primary resection that may have been performed (2). Linitis plastica-type gastric cancer tumors tend to infiltrate the submucosa and muscularis propria of the gastric wall, thus, superficial mucosal biopsies may be falsely negative, and detecting the extent of the spread and depth of the linitis plastica-type gastric cancer can be difficult endoscopically. The present report describes a patient with linitis plastica-type gastric cancer, in whom the characteristic morphological changes in the folds of the gastric body facilitated the determination of the spread and depth of tumor invasion. Written informed consent was obtained from the patient.

## Case report

**Figure 1:**
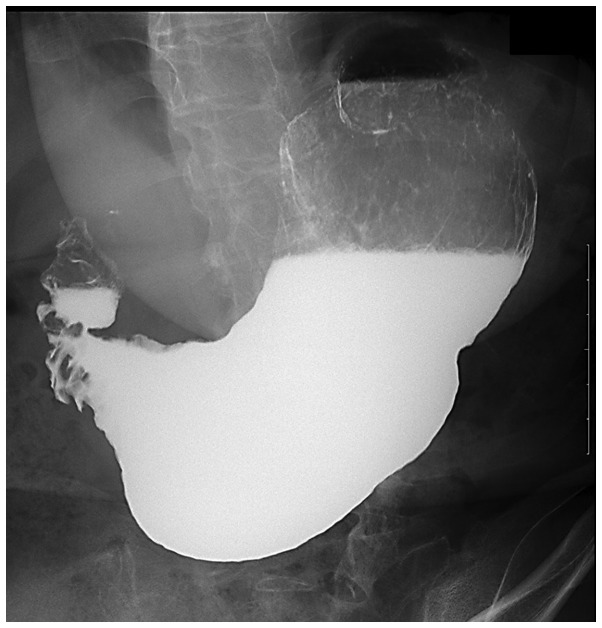
Stomach X-ray of the patient, indicating poor distension of the gastric body.

**Figure 2:**
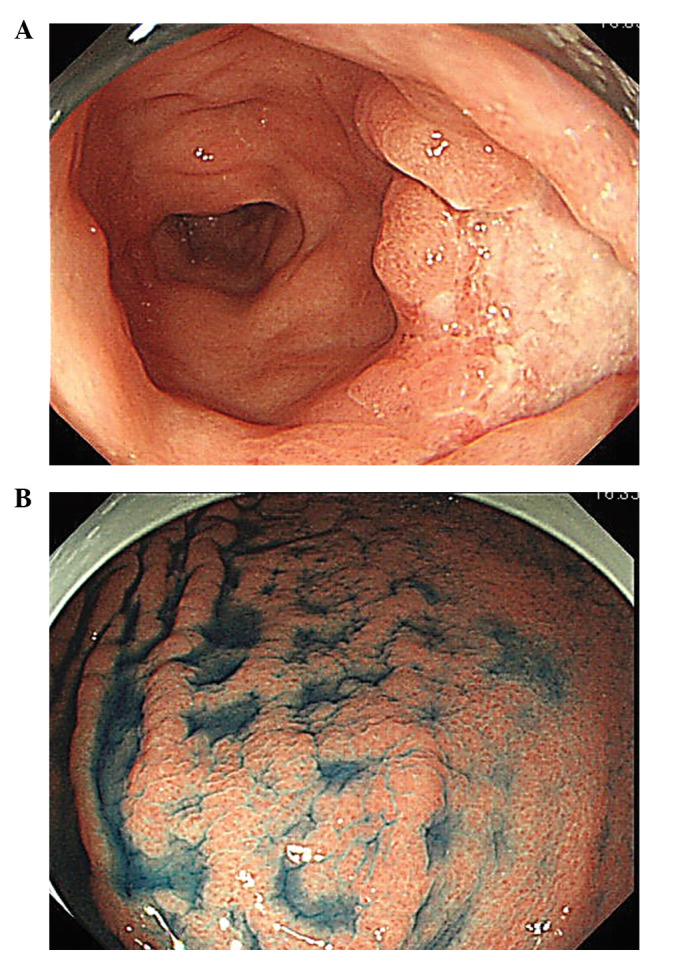
Endoscopic images of; (A) the primary depressed lesion at the posterior wall of the gastric antrum and (B) the crossed folds with a waffle-like appearance on the greater curvature of the upper gastric body.

**Figure 3:**
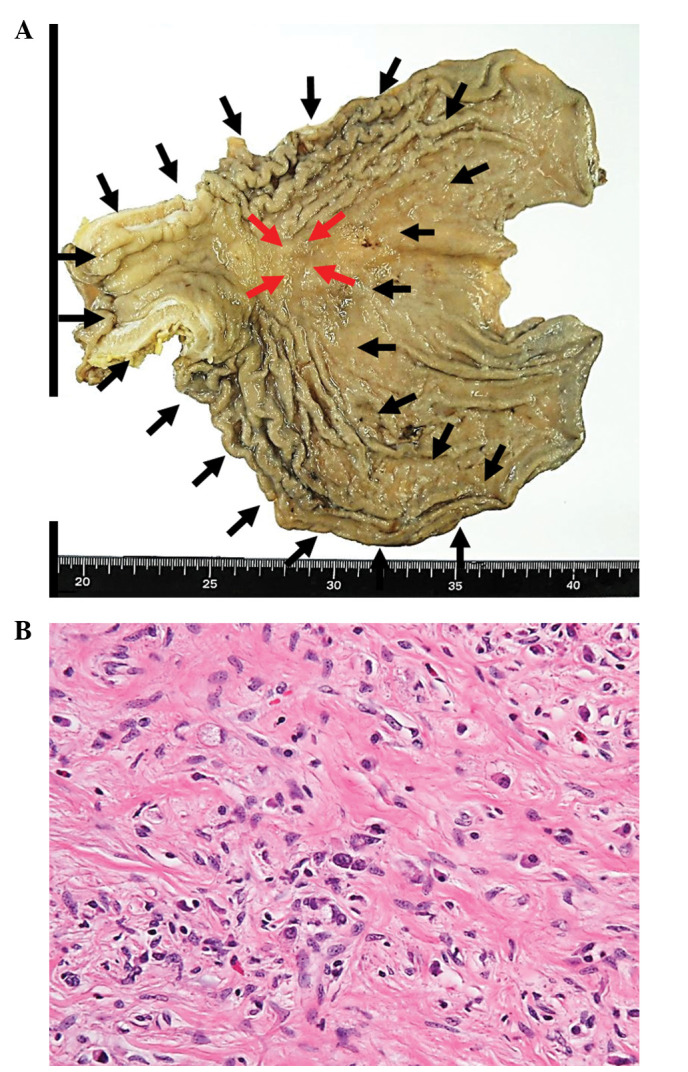
(A) Resected specimen. Red arrows indicate the primary depressed lesion and the black arrows indicate the areas of cancer invasion. (B) Microscopic image, revealing the primary lesion to be a poorly differentiated adenocarcinoma containing signet ring cells (stain, hematoxylin and eosin; magnification, ×400).

A 66-year-old female was admitted to Kagawa University Hospital (Kagawa, Japan) with the complaint of intermittent epigastric pain that was exacerbated by fasting. The patient had a history of hypertension and obstructive sleep apnea syndrome. Physical examination upon admission revealed no anemia (via conjunctival pallor examination), jaundice or pulmonary abnormalities. On palpation, the abdomen of the patient was soft and flat, with no areas of tenderness. Furthermore, pretibial edema was not observed and superficial lymph nodes were not palpable. Serum concentrations of the tumor markers, carcinoembryonic antigen and carbohydrate antigen 19-9, were within the normal ranges (<5 ng/ml and 0–37 U/ml, respectively). However, X-ray examination indicated reduced gastric distension, as well as deformation of the stomach, which exhibited a leather bottle-like appearance (Fig. 1). In addition, the lower gastric body demonstrated luminal narrowing and increased rigidity, with a depressed lesion (longest diameter, 20 mm) at the posterior wall of the gastric antrum and abdominal computed tomography revealed thickening of the antrum. No lymphadenopathy was observed. Additionally, endoscopy revealed an ulcerative lesion covered by a white necrotic substance on the posterior wall of the antrum (Fig. 2A) and severe luminal narrowing, with poor distension of the lower gastric body. The upper gastric body, however, demonstrated good extension when compared with the middle and lower gastric bodies. The folds of the gastric antrum were flexible, stretched smoothly, and crossed one another, resulting in a waffle-like appearance on the greater curvature of the upper gastric body (Fig. 2B). Analysis of biopsy specimens from the ulcerative lesion revealed a poorly differentiated adenocarcinoma containing signet ring cells, however, adenocarinoma was absent from biopsy specimens obtained from the abnormally crossed folds. Due to the morphological changes that occured in the gastric folds, creating the waffle-like appearance, it was determined that cancer cell invasion of the upper gastric body was likely, and a total gastrectomy was performed. The resected specimen revealed the wall thickening and crossing folds of the gastric body (Fig. 3A) that were previously observed by endoscopy. Microscopic examination revealed that cancer cells had spread throughout the upper gastric body and had infiltrated the vessels in the submucosa, predominately into the muscularis propria, and marginally into the serosa (Fig. 3B). Immunohistochemical examination revealed positive staining for MUC5AC and MUC6 (gastric marker mucins) and negative staining for MUC2 and CD10 (intestinal marker mucins), indicating gastric-type mucin expression. The final diagnosis, according to the Japanese Classification of Gastric Carcinoma (3), was T4aN3aM0, clinical stage IIIC advanced gastric cancer. The patient was discharged 17 days after surgery without complications and commenced three cycles of S-1 adjuvant chemotherapy (80 mg/day, days 1–28) for 16 weeks. Following three courses of chemotherapy for 16 weeks, treatment was terminated due to patient fatigue. The patient has survived and is without disease recurrance 14 months after surgery.

## Discussion

The Japanese Classification of Gastric Carcinoma has defined Type IV diffuse infiltrative gastric cancer as tumors lacking marked ulceration or raised margins, but with thickened and indurated gastric walls, and poorly defined margins (3). Type IV carcinoma, or scirrhous gastric carcinoma, is therefore a diffuse infiltrating adenocarcinoma, which is predominantly poorly differentiated and lacking a circumscribed lesion. Tumor involvement of the entire stomach wall results in a condition termed linitis plastica (4). Despite improved treatment outcomes for other types of gastric carcinoma in Japan, the prognosis of patients with linitis plastica remains particularly poor (5,6).

Endoscopic brush cytology and biopsy techniques have an overall sensitivity of 95–98% in the detection of gastric cancer (7,8). However, the accuracy of endoscopy ranges widely, depending on the gross tumor growth pattern and the anatomic location of the tumor (9), with a sensitivity of only 33–73% observed in linitis plastica patients (9–12). The predominant reason for the poor sensitivity of endoscopy in the detection of scirrhous gastric cancer is the healthy appearance of the mucosa.

Although the invasion and spread of linitis plastica-type gastric cancer is difficult to diagnose prior to surgery, the presence of depressed lesions and marginal changes in the gastric folds may be indicators for diagnosis. Endoscopic findings that are characteristic of scirrhous gastric cancer include poor distension of the gastric walls, morphological changes in the gastric folds and the presence of primary lesions (13). The differential diagnosis of thickened gastric folds includes Ménétrier’s disease, hypertrophic gastritis, malignant lymphoma, rare types of aberrant pancreas, syphilis and cytomegalovirus gastritis (14). Morphological changes in the gastric folds of linitis plastica patients include the presence of giant, swollen, straight, furrowed and crossed folds. These morphological changes are important for distinguishing linitis plastica from other diseases. Further diagnostic indicators of linitis plastica include poor distention of the stomach, and a leather bottle-like appearance as observed by gastrointestinal (GI) series, indicating that an upper GI series may be superior to endoscopic examination in the diagnosis and localization of these types of lesions (15). By contrast, certain linitis plastica-type gastric cancer patients do not demonstrate the typical deformity, poor distension, or irregular folds via upper GI series, however, exhibit focal alterations in infiltrated areas (16).

Linitis plastica is also characterized by poorly differentiated tumor cells that diffusely infiltrate the gastric wall, thus leading to reactive fibrosis (17). Early stage, undifferentiated [0-IIC morphological type (3)], linitis plastica-type gastric cancer is present in the mucosa of the gastric wall, progressing by diffuse infiltration into the submucosal layer prior to ulceration of the primary lesion, with the cancer cells extending beyond the fibrous tissue. In patients with mild fibrosis, the stomach distends well upon air insufflation, with the gastric body occasionally exhibiting a waffle-like appearance; these observations may be important in the accurate diagnosis of the spread of cancer cell invasion.

Recently, we performed a bloc biopsy, using the submucosal endoscopy mucosal flap to diagnose submucosal tumors (18–20). This technique lead to histopathological diagnosis without any complications, such as hemorrhage or dissemination of tumor. In the future, this method may exhibit potential diagnosis value for accurately diagnosing a linitis plastica-type gastric cancers.

In conclusion, accurately diagnosing the spread and depth of linitis plastica-type gastric cancer requires examination of the morphological changes in the gastric folds, as well as examination of the primary depressed lesion. The use of novel methods, such as bloc biopsy, may improve the accuracy of linitis plastic-type gastric cancer diagnosis.
